# Anti-CTLA-4 antibodies in cancer immunotherapy: selective depletion of intratumoral regulatory T cells or checkpoint blockade?

**DOI:** 10.1186/s13578-018-0229-z

**Published:** 2018-04-18

**Authors:** Fei Tang, Xuexiang Du, Mingyue Liu, Pan Zheng, Yang Liu

**Affiliations:** 1 Division of Immunotherapy, Institute of Human Virology and Department of Surgery, University of Maryland School of Medicine, 725 W Lombard Street, Baltimore, MD 21201 USA; 2 OncoImmune, Inc., 9430 Key West Avenue, Rockville, MD 20850 USA

**Keywords:** CTLA-4, Ipilimumab, B7-CTLA-4 interaction, Regulatory T cells, Tumor microenvironment, Cancer immunotherapy

## Abstract

Antibodies to human CTLA-4 have been shown to induce long-lasting protection against melanoma. It is assumed that these antibodies cause tumor rejection by blocking negative signaling from the B7-CTLA-4 interactions to enhance priming of naïve T cells in the lymphoid organs. Recently, we reported that anti-CTLA-4 antibody Ipilimumab effectively induces tumor rejection in vivo although it blocks neither B7 transendocytosis by CTLA-4 nor CTLA-4 binding to immobilized or cell-associated B7. Using genetic model in which the anti-CTLA-4 antibodies are unable to engage more than 50% of CTLA-4, we demonstrated that saturating binding of CTLA-4 is not necessary for tumor rejection. Our results argue against B7-CTLA-4 blockade as the mechanism of action for the clinically effective Ipilimumab. Moreover, Ipilimumab induces tumor rejection even in the absence of de novo T cell priming in the lymphoid organs. Thus, our data are inconsistent with key provisions of the prevailing hypothesis on mechanism of action by anti-CTLA-4 antibodies. Furthermore, anti-CTLA-4 antibodies effectively induce depletion of regulatory T (Treg) cells in tumor microenvironment but not in the peripheral lymphoid organs, which is strictly dependent on Fc receptor on host cells. Based on these data and other recent publications on the subject, we propose that anti-human CTLA-4 antibodies induce tumor rejection by selective depletion of Tregs in the tumors rather than blockade of B7-CTLA-4 interaction in lymphoid organs.

In 2011, the FDA approved the first anti-CTLA-4 antibody, Ipilimumab (trademark name YERVOY^®^), for the treatment of melanoma. Ipilimumab has demonstrated substantial and durable therapeutic effects, and is now undergoing clinical trials in treating many other cancers. According to the checkpoint blockade hypothesis [1], anti-CTLA-4 antibodies cause tumor rejection by promoting priming of naïve T cells through blocking the inhibitory B7-CTLA-4 signaling in peripheral lymphoid organs (Fig. 1). However, this prevailing hypothesis has not been rigorously tested. Ipilimumab was selected according to its ability in blocking the interaction between anchored CTLA-4 and soluble B7 molecules [2]. However, since B7 ligands are co-stimulatory molecules expressed on cell surface, it remains to be tested whether Ipilimumab can effectively block the B7-CTLA-4 interactions under physiological conditions.

**Fig. 1:**
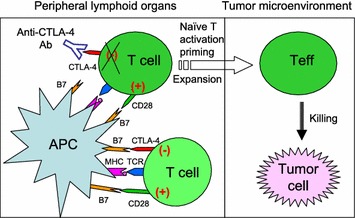
The prevailing view: CTLA-4 checkpoint blockade results in tumor immunity. Activation of T cells requires two signals. One is the binding of the T cell receptor (TCR) to the MHC-antigen peptide complex presented by antigen presenting cells (APCs) (signal 1). The other one is the binding of B7 molecules (B7-1 or B7-2) to the co-stimulatory (+) molecule CD28 on the surface of T cells (signal 2). With higher affinity than CD28, inhibitory (−) CTLA-4 binds to B7 ligands on APCs and provides a brake  for T cell activation. Anti-CTLA-4 antibodies were proposed to release brakes of naïve T cells and allow them to be activated in the lymphoid organs and then migrate to tumors to cause tumor rejection

Recently, we compared multiple anti-CTLA-4 monoclonal antibodies (mAbs) for their abilities to block B7-CTLA-4 interactions under various settings [3]. We found that when B7 molecules were immobilized on solid phases (such as when B7-1 or B7-2 was coated on ELISA plates or expressed on cell surface), Ipilimumab was unable to block the interaction between B7 and CTLA-4 [3]. On the contrary, L3D10, one anti-CTLA-4 antibody generated in our lab [4, 5], can block the in vitro interactions between various forms of CTLA-4 and B7 molecules [3]. Similar trend was observed when CTLA-4-mediated transendocytosis was measured. Consistent with this finding, we found that L3D10, but not Ipilimumab, significantly inhibited CTLA-4-mediated downregulation of B7 on the surface of splenic dendritic cells in *CTLA4*^h/h^ humanized mice and human CD34^+^ stem cell reconstituted mice, providing in vivo evidence that Ipilimumab is ineffective in blocking B7-CTLA-4 interaction under physiological conditions [3].

Despite the differences in blocking B7-CTLA-4 interaction, L3D10 and Ipilimumab are comparable in inducing anti-tumor activity [3]. In addition, the fully humanized L3D10 clones, HL12 and HL32, which lose the ability to block B7-CTLA-4 interaction, remain fully active in inducing tumor rejection. Together, the above data demonstrate that blockade of B7-CTLA-4 interaction is unnecessary for immunotherapeutic effect of anti-CTLA-4 antibodies. In *CTLA4*^h/m^ heterozygous mice, which express mouse and human CTLA-4 molecules in a codominant manner, anti-human CTLA-4 antibodies are unable to engage more than 50% of CTLA-4 as the remaining 50% of the molecules are of mouse origin and thus lack reactivity to antibodies originally made in mice [3]. However, all anti-CTLA-4 antibodies caused robust tumor rejection in *CTLA4*^h/m^ mice. Therefore, even for blocking antibodies, their ability to completely block B7-CTLA-4 interaction is not required for effective tumor immunotherapy.

Finally, we reasoned that since antibody treatment is initiated after T cell priming has already taken place in the lymphoid environment, it is possible that anti-CTLA-4 antibodies may promote tumor rejection even if their effect in de novo T cell priming is abrogated. We tested if Ipilimumab can cause tumor rejection if de novo T cell priming is shut down by complete blockade of B7 by anti-B7 antibodies. Our results demonstrated that while anti-B7 antibodies effectively blocked de novo T cell priming in lymphoid organs, Ipilimumab remained fully active in causing tumor rejection in the presence of saturating B7 blockade [3]. These data refute the idea that anti-CTLA-4 antibodies cause tumor rejection by promoting T cell priming in the lymphoid organs.

What then is the primary mechanism of anti-CTLA-4 antibody induced tumor rejection? Several groups have established that anti-mouse CTLA-4 antibodies induced tumor rejection through selective depletion of regulatory T (Treg) cells in the tumor microenvironment [6–8]. By showing selective Tregs depletion in tumor microenvironment by anti-CTLA-4 antibodies as well as the absolute requirement of Fc receptor (FcR) in Ipilimumab-induced tumor rejection [3], our work demonstrates that the effector mechanism of anti-human CTLA-4 antibodies is similar to that reported by anti-mouse CTLA-4 antibodies (Fig. 2).

**Fig. 2:**
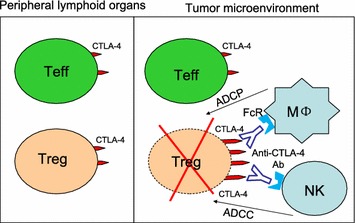
New concept: selective depletion of Tregs in the tumor microenvironment results in tumor immunity. Higher levels of CTLA-4 on intratumoral Tregs allow their selective depletion by anti-CTLA-4 antibodies, perhaps through antibody-dependent cellular phagocytosis (ADCP) by macrophages and/or antibody-dependent cellular cytotoxicity (ADCC) by NK cells. Blocking of B7-CTLA-4 interaction is not required for effective depletion of intratumoral Treg cells

Therefore, rather than focusing on enhancing the blocking of B7-CTLA-4 interaction, other approaches on promoting local intratumoral Tregs clearance should be attempted in order to optimize the therapeutic effect of anti-CTLA-4 antibodies.

## Abbreviations

APC: antigen presenting cell
CTLA-4: cytotoxic T-lymphocyte associated protein 4
FcR: Fc receptor
MHC: major histocompatibility complex
NK: natural killer
TCR: T cell receptor
Teff: effector T cells
Treg: regulatory T cells
